# SLC16A1 Inhibits Ferroptosis and Promotes the Progression of Head and Neck Squamous Cell Carcinoma

**DOI:** 10.7150/jca.110217

**Published:** 2025-03-29

**Authors:** Huaiyuan Zong, Luyao Teng, Lifang Chen, Jianxin Qiu, Chunhui Tian

**Affiliations:** 1 Department of Otorhinolaryngology Head and Neck Surgery, Suzhou Hospital Affiliated of Anhui Medical University, Suzhou, 234000, China.; 2 Department of Biochemistry & Molecular Biology, School of Basic Medicine, Anhui Medical University, Hefei, 230032, China.; 3 Department of College of Marine Life Sciences, Ocean University of China, Qingdao, 266003, China.; 4 Department of Otorhinolaryngology Head and Neck Surgery, The First Affiliated Hospital of Anhui Medical University, Hefei, 230022, China.

**Keywords:** HNSCC, SLC16A1, ferroptosis, tumor growth

## Abstract

The solute carrier family 16 member 1 (SLC16A1) gene demonstrates abnormally elevated expression levels in a variety of human malignant tumors, and it is pivotal in tumor initiation and progression. Nonetheless, the precise mechanisms through which this gene operates in head and neck squamous cell carcinoma (HNSCC) need to be elucidated. This study integrated bioinformatics analysis with clinical patient samples to elucidate that the mRNA and protein levels of SLC16A1 were significantly upregulated in patients with HNSCC, which was closely associated with poor patient prognosis. In addition, through the construction of stable SLC16A1 knockdown and overexpression models in HNSCC cells along with *in vitro* and *in vivo* experiments, the study comprehensively illuminated the pivotal role of SLC16A1 in promoting the proliferation, migration, and invasiveness of HNSCC cells, as well as enhancing their resistance to ferroptosis. *In vitro* experimental results demonstrated that when SLC16A1 was knocked down, the proliferation, migration, and invasion capabilities of HNSCC cell lines were significantly reduced and the extent of RAS-selective lethal 3-induced lipid peroxidation increased compared with control cells. Conversely, HNSCC cell lines overexpressing SLC16A1 exhibited enhanced proliferation, migration, and invasion capabilities, accompanied by lower levels of lipid peroxidation. *In vivo* experiments further corroborated the pivotal role of SLC16A1 in promoting HNSCC tumor growth. Our research findings indicate that SLC16A1 acts as an oncogene in HNSCC, and that abnormally high expression of SLC16A1 significantly accelerates the development and progression of HNSCC by conferring resistance to ferroptosis.

## Introduction

Head and neck squamous cell carcinoma (HNSCC) is one of the most common types of cancer. HNSCC, which primarily originates from the mucosal epithelial tissues of the oral cavity, pharynx, nose, and larynx, is known for its high invasiveness and propensity for metastasizing to cervical lymph nodes 1, 2. There were 54,540 new cases of HNSCC in the United States alone, with 11,580 deaths attributed to the disease, according to 2023 statistics 3. The main risk factors include excessive use of tobacco products, heavy alcohol consumption, human papillomavirus infection, and acid reflux into the throat 4-6. Despite significant advancements in diagnostic and treatment methods, the five-year survival rate for patients with HNSCC has not shown any marked improvement over the past 30 years, remaining at approximately 50% 7, 8. Therefore, the search for new therapeutic targets for HNSCC remains an urgent need.

The concept of ferroptosis was first proposed in 2012 9. A novel form of programmed cell death driven by iron-dependent lipid peroxidation, ferroptosis is distinguished from traditional modes of cell death such as apoptosis, necrosis, and autophagy 9, 10. Ferroptosis exhibits unique characteristics both morphologically and mechanistically; morphologically, it is primarily characterized by mitochondrial shrinkage, increased mitochondrial membrane density, and a reduction in mitochondrial cristae 11. Mechanistically, the glutathione peroxidase 4/glutathione (GSH) system is considered a critical defense mechanism against ferroptosis in cells 12. Lipid peroxidation is the core reaction of ferroptosis, a process that leads to the oxidative degradation of lipids, resulting in the formation of peroxides and hydroperoxide derivatives 13. The primary products of lipid peroxidation are lipid hydroperoxides. Among the secondary products, malondialdehyde (MDA) is considered to have the highest mutagenicity. Known inducers of ferroptosis include erastin and RAS-selective lethal 3 (RSL3), while antioxidants such as liproxstatin-1 (Lip-1), can inhibit the process of ferroptosis 14. Increasing research evidence indicates the existence of a close relationship between ferroptosis and the development and progression of tumors 15-17; therefore, investigating the role of ferroptosis in HNSCC holds significant importance.

The solute carrier family 16 member 1 (SLC16A1), also known as monocarboxylate transporter 1, is located on the short arm of human chromosome 1 18. The protein encoded by this gene facilitates the rapid transport of certain monocarboxylates, such as pyruvate or lactate, across the cell membrane, and it plays a key role in various physiological activities of the cell, including energy metabolism and signaling 19, 20. Notably, SLC16A1 exhibits high expression in various types of tumors, and it is considered to play an important role in the process of tumor metastasis 21-26. For example, SLC16A1 can interact directly or indirectly with components of the NF-κB signaling pathway, thereby enhancing the survival ability and metastatic potential of tumor cells 23. In addition, studies have shown that SLC16A1 inhibits ferroptosis, thus promoting the development of liver cancer 27. However, research regarding the function of SLC16A1 as well as its mechanisms of action in HNSCC is relatively limited. Therefore, further exploration of the specific impacts of SLC16A1 in HNSCC and its mechanisms of action is urgently needed to identify novel therapeutic targets for this disease.

## Materials and methods

### Reagents and antibodies

RSL3, Lip-1 were sourced from Med Chem Express (NJ, USA). Dimethyl sulfoxide (DMSO) was purchased from Sigma-Aldrich (MO, USA). Puromycin was obtained from Solarbio (Beijing, China). The antibodies used as follows: SLC16A1 (Cat#20139-1-AP) was purchased from Proteintech (Wuhan, China), GAPDH (Cat#5174s) was purchased from Cell Signaling Technology (MA, USA), β-actin (Cat#A1978) was obtained from Sigma-Aldrich.

### Cell lines and cell culture

The human laryngeal squamous cell carcinoma (TU177), human normal squamous epithelial cells (NOK), human laryngeal cancer cells (TU212), human pharyngeal squamous cancer cells (FaDu), and LIU-LSC-1 cells have been described previously 28, 29, human tongue squamous cell carcinoma (HN6) was sourced from Otwo Biotech (Shenzhen, China). All cell lines were cultured in DMEM Medium (Biochannel, Nanjing, China), enriched with 10% fetal bovine serum (Lonsera, Suzhou, China) and 1% penicillin-streptomycin (Beyotime, Shanghai, China), to ensure optimal growth conditions.

### Clinical specimens

Over the span of 2022 to 2024, Suzhou Hospital Affiliated to Anhui Medical University meticulously collected 12 pairs of HNSCC samples along with their corresponding adjacent normal mucosal (ANM). Prior to the surgical procedure, all recruited patients were in a state of not receiving any form of anti-tumor treatment, including chemotherapy, radiotherapy, or other related therapies. The use of all collected tissue samples was approved by the Research Ethics Committee of Suzhou Hospital Affiliated to Anhui Medical University. Comprehensive patient information was appended in **Supplementary Table S1**.

### Lentivirus infection

The lentiviruses infecting the HNSCC cells (TU177 and HN6) were constructed and synthesized by General Biol (Chuzhou, China) using the VP013 or PLVX vector, including lentiviral shRNA expression vectors targeting SLC16A1 and control scrambled shRNA (shSc) for non-specific interference; lentiviral plasmid expressing SLC16A1 cDNA, and the empty plasmid. The target sequences were shown as follows: shSc: 5′-TTCTCCGAACGTGTCACGT-3′; shSLC16A1^1^: 5′-CAAAGAGATTGAAGGTATATT-3′; shSLC16A1^2^: 5′-ATCAGTCTTCCAAACAATTAA-3′. The lentiviral particles were introduced into the targeted cell lines (TU177 and HN6) in strict adherence to the manufacturer's recommended protocol. Following a week-long selection period using puromycin, which served to enrich for cells successfully transduced with the lentiviral constructs, the infection efficiency was rigorously assessed using western blotting analysis to detect protein expression.

### Western blotting

The previously detailed methodology was adhered to for conducting the western blotting analysis 30. In summary, RIPA lysis buffer (Beyotime) was used for extracting cellular or tissue proteins. Subsequently, the lysates were separated by NuPAGE 4-12% Bis-Tris gels (Life Technologies, CA, USA) and then transferred onto PVDF membranes (Millipore, MA, USA). After blocking with 5% non-fat milk for 1 h, the membranes were incubated overnight at 4°C with specific primary antibodies, followed by incubation with secondary antibodies at room temperature for 1 h. Chemiluminescence was then used to visualize the specific protein bands on the membrane.

### Quantitative real-time polymerase chain reaction (qRT-PCR)

As mentioned earlier 30, 31, the total RNA was extracted from tissues or cells utilizing the Trizol Reagent (Genesand, Beijing, China), and reverse transcribed into complementary DNA (cDNA) using the First-strand cDNA Synthesis Mix for qPCR (Genesand). qRT-PCR was conducted employing SYBR Premix Ex TaqTM II (TaKaRa, Kyoto, Japan), and the amount of RNA was analyzed using LightCycler® 96 (Roche, Switzerland). The primer sequences (Sangon Biotech, Shanghai, China) were shown as follows: SLC16A1: F: 5′-TCAGGCTGTGGCTTGATTGC-3′, R: 5′-GCCAATGGTCGCCTCTTGTAGAA-3′; β-actin: F: 5′-ATCGTCCACCGCAAATGCTTCTA-3′, R: 5′-AGCCATGCCAATCTCATCTTGTT-3′.

### Cell proliferation assay

Distribute the specified cells into a 96-well plate at a precise density of 1000 cells per well. Following the manufacturer's product instructions, use the Cell Counting Kit-8 (CCK8) kit (Beyotime) to quantify the proliferation rate of the cells. In summary, add 10 μl of CCK-8 solution to each well of the 96-well plate, incubate in the dark at 37°C for 1 h, and then measure the optical density (OD) value at 450nm.

### Colony formation assays

Seed 1×10^3^ cells per dish into 10 cm culture dishes, and incubate under the conditions of 37°C and 5% CO_2_ for 14 days. Following this, fix the cells with 4% paraformaldehyde for 30 min. Finally, stain the cells with 0.1% crystal violet for 30 min. Afterwards, take photographs and calculate the number of colonies formed.

### Cell viability assay

In a 96-well plate, 10,000 cells were seeded per well. Once the cells had adhered, ferroptosis inducer RSL3 was applied for 24 h. Subsequently, CCK8 assay was performed to measure OD value at 450nm, and results were analyzed as previously outlined 30.

### Wound healing assay

In a wound healing assay, cells were initially seeded in a 6-well plate and incubated overnight under suitable conditions to form a confluent monolayer. Following this, a sterile 200 microliter pipette tip was used to draw a straight line across the cell monolayer, creating a gap that simulates a wound. Subsequently, microscopic images were taken at 0 h and again at 24 h, in the same field of view, to track and evaluate the migration of cells into the scratch area.

### Cell migration and invasion assays

Transwell assays employed 24-well chambers (Corning, NY, USA). 20,000 cells in 200 μl serum-free DMEM were seeded above, while 500 μl 10% FBS medium was placed below. Chambers were incubated at 37°C with 5% CO_2_ for 24 h. Migration assays lacked Matrigel (Corning), whereas invasion assays featured Matrigel coating. Then, top-side cells were removed, and those on the underside were fixed with 4% paraformaldehyde and stained with crystal violet. Quantification of migration and invasion was achieved by counting cells in 10 random fields at 200× magnification under a microscope.

### Lipid reactive oxygen species (L-ROS) assay

L-ROS was determined with flow cytometry using C11 BODIPY (581/591) (ThermoFisher Scientific, CA, USA). Cells were seeded at a density of 1×10^6^ per well in a six-well dish, grown overnight, and then treated with RSL3 or DMSO for 24 h. Then, 2 µm of C11-BODIPY was added, and the samples were incubated for 25 min at 37 °C, 5% CO_2_, while being protected from light. Cells were washed twice with PBS to remove excess labeling mixture and harvested by trypsinization, transferred to a 1.5 ml microfuge tube, pelleted, and resuspended in 0.4 ml of PBS. The fluorescence intensities of cells per sample were determined by the green and red fluorescent signal channels. A minimum of 10,000 cells was analyzed for each sample.

### MDA assay

The concentration of MDA, a lipid peroxidation product, was assessed in cells using a Lipid Peroxidation MDA Assay Kit (Beyotime), following the manufacturer's instructions. Briefly, cells were plated in 6 cm dishes (at a density of approximately 2×10^6^ cells per dish) and treated with RSL3 or DMSO for 24 h. Subsequently, the cells were lysed using a cell lysis solution, and the supernatant was collected by centrifugation. The protein concentration in these cell samples was quantified using the BCA kit (Beyotime). To prepare the samples for analysis, they were subjected to a boiling water bath for 15 min, followed by another centrifugation at 1000 g for 10 min to collect the supernatant. Afterwards, the supernatant was aliquoted into a 96-well plate, and the absorbance was measured at a wavelength of 532 nm. The MDA levels were then calculated using standard curve analysis.

### GSH assay

According to the instructions provided by the GSH and GSSG Assay Kit (Beyotime), the designated cells were seeded into 6-cm cell culture dishes at an appropriate density and incubated overnight. Subsequently, RSL3 was added, and the cells were exposed to it for 24 h before being collected. The concentration of reduced GSH was calculated using the method described previously 30, 32.

### Transmission electron microscopy (TEM)

Cells were plated in 15 cm dishes and treated with RSL3 or DMSO for 24 h, fixed with 4% PFA for 15 s, followed by fixation in 2.5% glutaraldehyde solution. After washing three times with Millonig's phosphate buffer, the cells were incubated with osmium tetroxide, and then washed three times again with Millonig's phosphate buffer. The samples underwent dehydration, permeabilization, embedding, and were sliced into 70 nm ultrathin sections using an ultramicrotome (Leica UC7). These sections were then lead-stained and imaged with a transmission electron microscope (JEM1400, Japan).

### Animal experiments

The BALB/c-nude mice used in this study were all purchased from GemPharmatech (Nanjing, China). Throughout the animal experimentation, we strictly adhered to the guidelines for the management and use of experimental animals set by the Animal Center of Anhui Medical University. We also obtained comprehensive approval from the Animal Ethics Committee of Anhui Medical University, ensuring that all experimental procedures complied with ethical standards. In the *in vivo* xenograft experiment, nude mice were randomly allocated into multiple cohorts, each consisting of 3 mice. Subcutaneous injections of 200 μl suspensions containing 5 × 10^6^ cells in DMEM basal medium were administered in the axillary region of each mouse. Tumor growth was assessed every three days, with volumes calculated using the formula: Volume = (length × width^2^) / 2 (mm³).

### Immunohistochemistry (IHC) analysis

Firstly, the tumor tissue was fixed in formalin, followed by paraffin embedding and sectioning. These sections were then incubated with selected primary antibodies overnight at 4°C. After the incubation, the sections underwent multiple washes and were further incubated with secondary antibodies conjugated with horseradish peroxidase. Finally, a DAB solution provided by Beyotime was used for the color development reaction, completing the entire IHC detection process.

### Bioinformatics analysis

The mRNA expression data of HNSCC patients with corresponding clinical information and somatic mutation data were downloaded from The Cancer Genome Atlas (TCGA, https://portal.gdc.cancer.gov/) database. When performing prognostic analysis, we chose to exclude cases with a survival time ≤30. A cohort of LSCC tissues (57 samples) and paired adjacent normal mucosa tissues (57 samples) was download from GSE127165(https://www.ncbi.nlm.nih.gov/geo/query/acc.cgi?acc=GSE127165). The corresponding ferroptosis-related genes were downloaded from FerrDb (http://www.zhounan.org/ferrdb/current/), a web-based consortium that provided a comprehensive and up-to-date database for ferroptosis markers, their regulatory molecules and associated diseases. The gene lists from the three datasets were provided in **Supplementary Table S2**. For each analysis, statistical significance was set at *P*<0.05. We divided TCGA-HNSCC patients into two groups by the gene expression. To evalute two groups compare the survival differences between low-risk and high-risk groups, Kaplan-Meier survival analysis was used by “survival” and “survminer” R packages. Univariate Cox regression and multivariate Cox regression analysis were used to evaluate whether gene expression was an independent prognostic factor.

### Statistical analysis

Group differences were examined using either the two-tailed Student's t-test or One-way ANOVA, depending on the nature of the data, with the GraphPad Prism 6.0 software. Statistical significance was determined based on a p-value threshold of < 0.05. Specifically, levels of significance were designated as follows: *P < 0.05, **P < 0.01, ***P < 0.001, and ****P < 0.0001. All the results were expressed as mean values with their corresponding standard deviations (mean ± SD). A p-value < 0.05 was interpreted as indicating a statistically significant difference between groups.

## Results

### Bioinformatics analysis for screening target genes related to HNSCC

**Figure 1:**
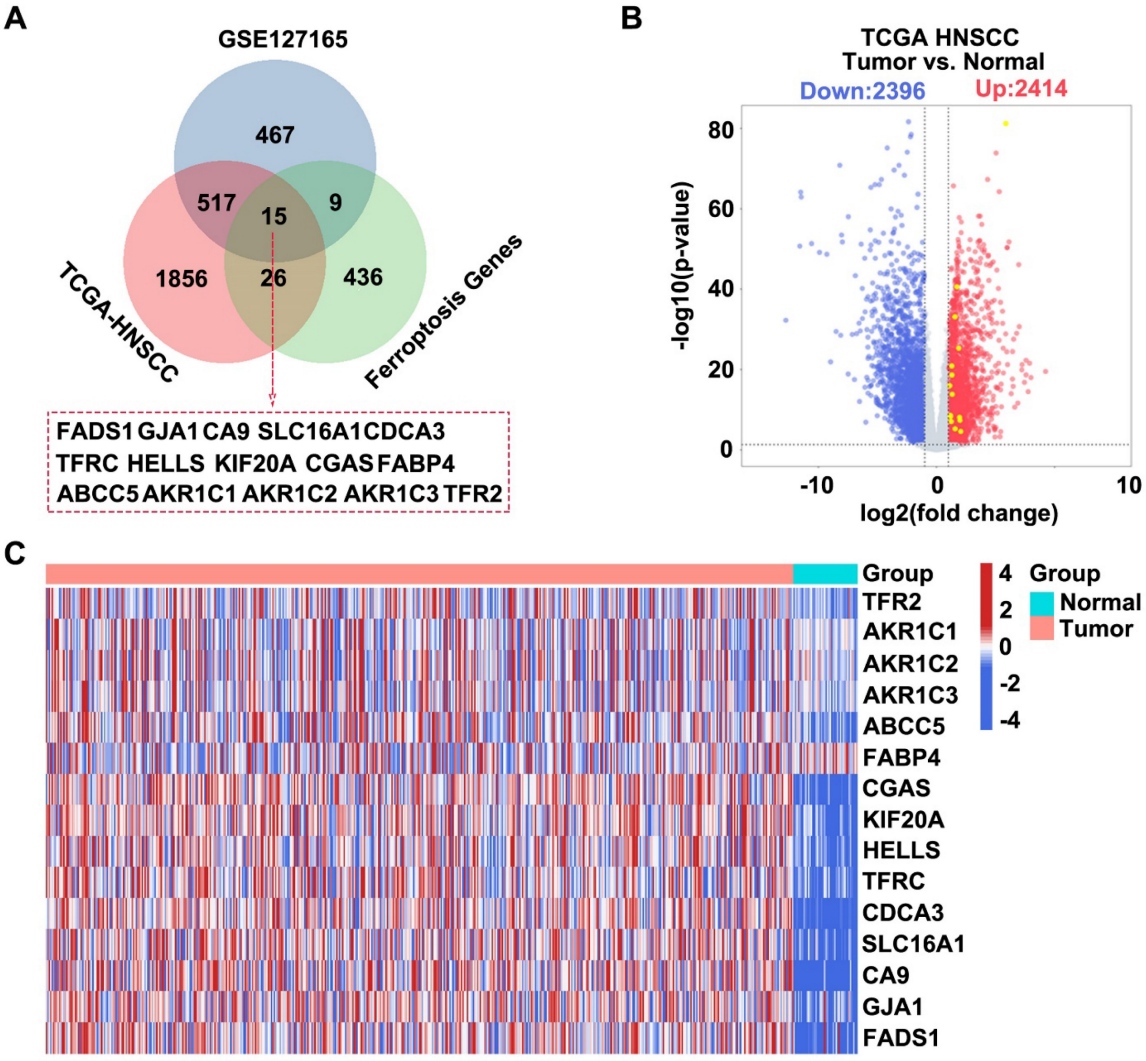
** Screening HNSCC-related target genes based on the TCGA GSE127165 FerrDb database.** (A) Venn diagram showing the intersection of the three datasets. Dataset 1 represents upregulated DEGs in GSE127165, Dataset 2 represents upregulated DEGs in HNSCC from TCGA, and Dataset 3 represents ferroptosis-related genes. (B) Construct a volcano plot for DEGs in HNSCC utilizing data from TCGA database. (C) Heatmap showing the expression profiles of the 15 intersecting genes.

We integrated information from three databases and constructed a Venn diagram to visualize their overlapping components to identify potential target genes associated with HNSCC. These databases included differentially expressed genes (DEGs) upregulated in HNSCC samples from TCGA, upregulated DEGs from the GSE127165 dataset, and known ferroptosis-related genes. We successfully identified 15 target genes through this integrative analysis approach **(Figure 1A)**. Subsequently, we used data from the TCGA-HNSCC database to generate a volcano plot that displayed significantly DEGs in HNSCC samples, including 2,414 upregulated and 2,396 downregulated genes **(Figure 1B)**. Finally, based on the information from the TCGA-HNSCC database, we created a heatmap for the 15 intersecting genes, which compared the gene expression patterns between patients with HNSCC and healthy controls **(Figure 1C)**.

### SLC16A1 is associated with poor prognosis in HNSCC

**Figure 2:**
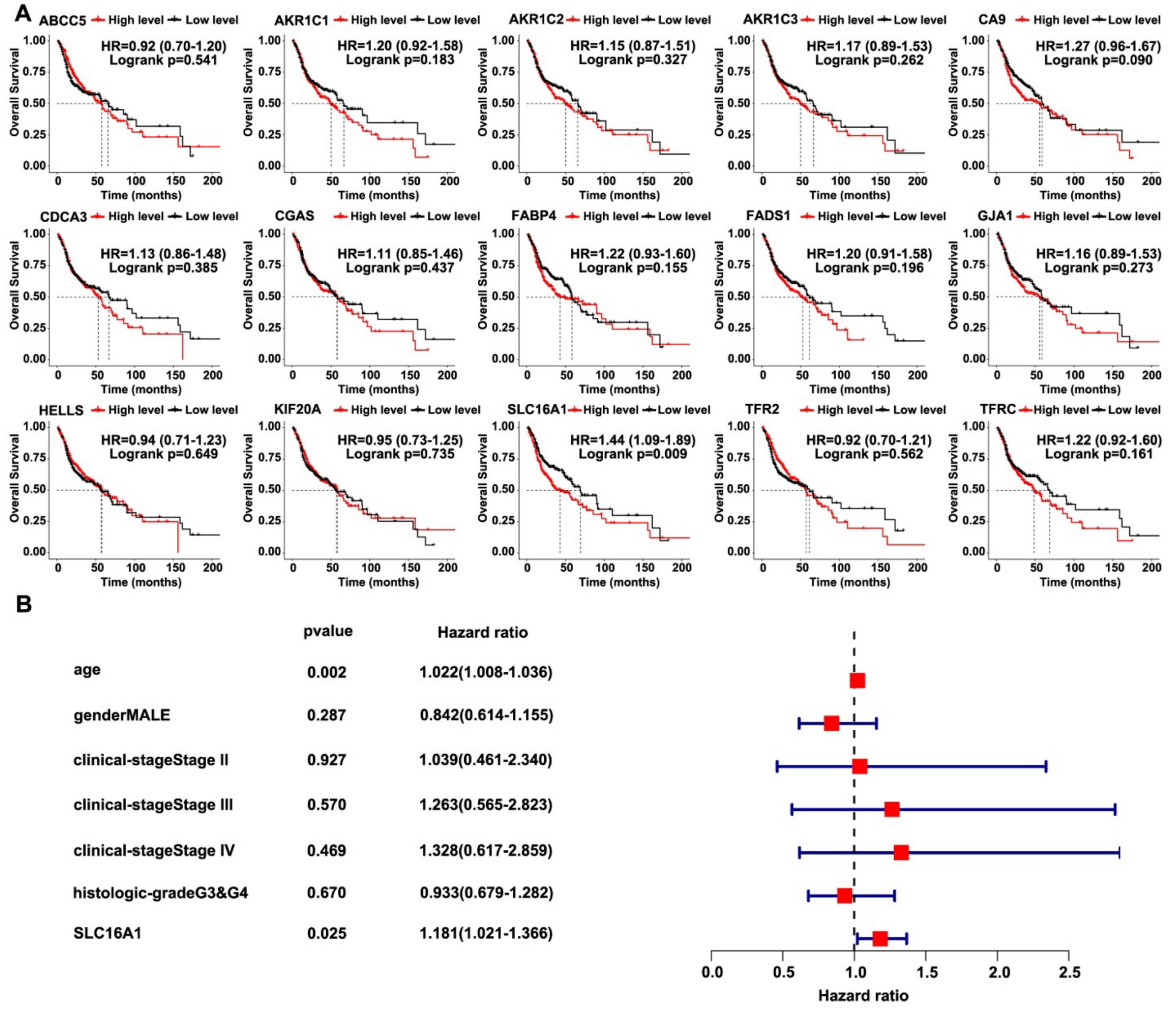
** SLC16A1 is associated with poor prognosis in HNSCC.** (A) Kaplan-Meier curves for overall survival of HNSCC patients based on the expression levels of 15 genes in the TCGA database. High and low expression groups were stratified by the median expression level. (B) Univariate Cox regression analysis for SLC16A1 in HNSCC patients.

We used the TCGA-HNSCC database to analyze the correlation between the survival of patients and the 15 intersecting genes in order to further screen for target genes related to HNSCC. Notably, only SLC16A1 indicated a poorer prognosis for patients with HNSCC** (Figure 2A)**. Subsequently, we conducted univariate Cox regression analysis using the TCGA-HNSCC database, which confirmed the association between SLC16A1 and adverse prognosis in patients with HNSCC** (Figure 2B)**.

### SLC16A1 is significantly elevated in head and neck tumors

**Figure 3:**
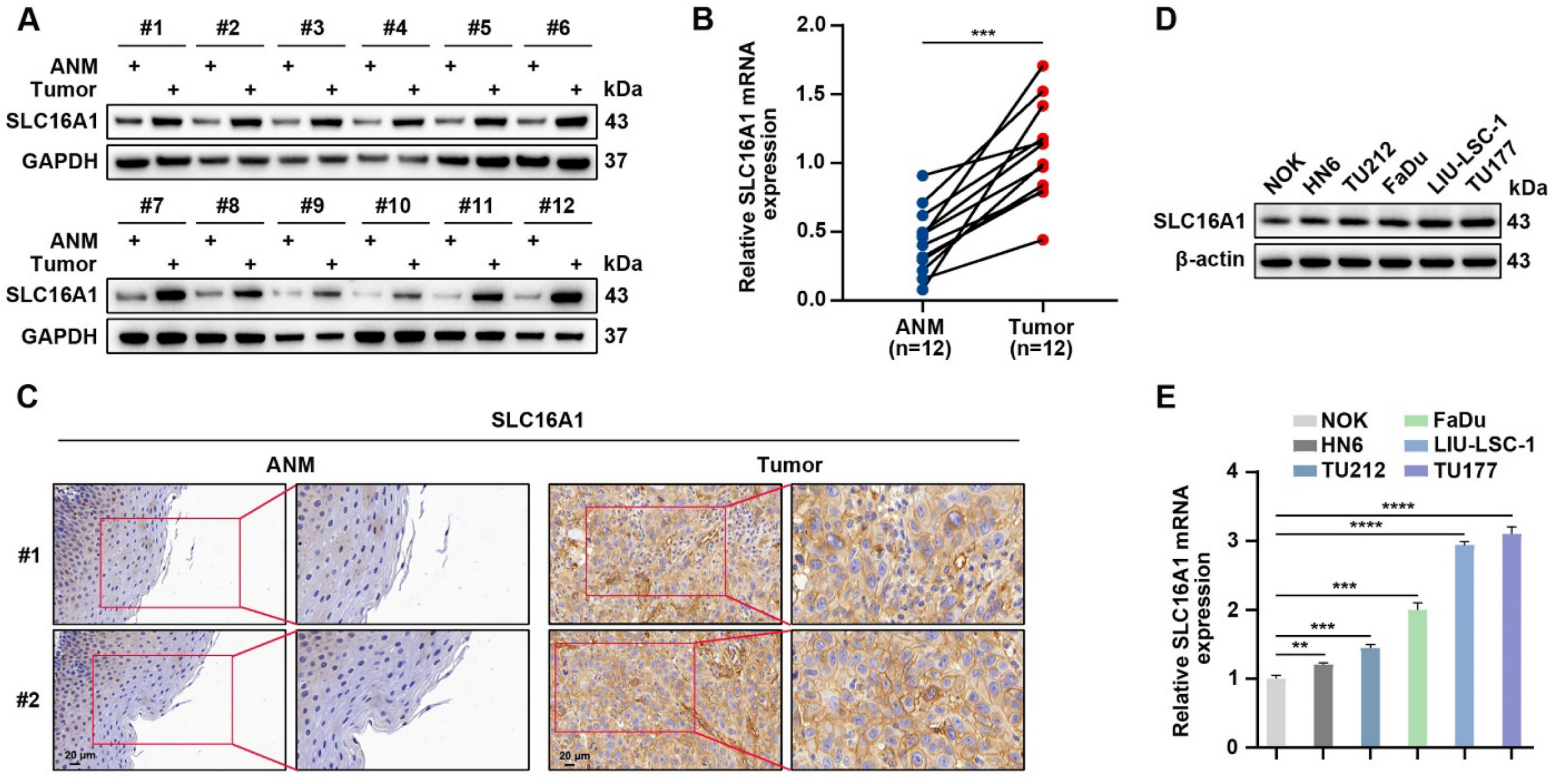
** SLC16A1 is significantly elevated in HNSCC.** (A and B) Western blotting (A) and qRT-PCR (B) analyses of SLC16A1 expression levels in 12 pairs of HNSCC tumor tissues and control ANM tissues. (C) Representative IHC images of SLC16A1. Scale bar, 20 μm. (D and E) Protein expression levels (D) and mRNA expression levels (E) of SLC16A1 in NOK cells and five HNSCC cell lines. Error bars indicate mean±SD of triplicate samples. **P<0.01; ***P<0.001; ****P<0.0001.

SLC16A1 has been demonstrated to exhibit high expression levels in multiple types of malignancies; however, its expression profile in HNSCC remains unclear. We conducted a comprehensive analysis using a suite of molecular biological techniques in order to elucidate the expression characteristics of SLC16A1 in HNSCC. Specifically, we used western blotting, qRT-PCR, and IHC to quantitatively evaluate the protein and mRNA expression levels of SLC16A1 in 12 pairs of HNSCC tissue samples and their corresponding adjacent normal tissues. Notably, in the HNSCC tissue samples, the protein and mRNA expression levels of SLC16A1 were significantly higher compared with those in the corresponding adjacent normal tissues **(Figure 3A-C)**. In addition, we used western blotting and qRT-PCR to assess the protein and mRNA expression levels of SLC16A1 in five HNSCC cell lines and NOK cells. Compared with the SLC16A1 in the NOK cells, the SLC16A1 in all of the HNSCC cell lines showed significantly upregulated expression levels **(Figure 3D-E)**. In summary, these experimental data collectively indicate that SLC16A1 is significantly upregulated in both HNSCC tissues and cell lines.

### SLC16A1 promotes HNSCC cell proliferation

**Figure 4:**
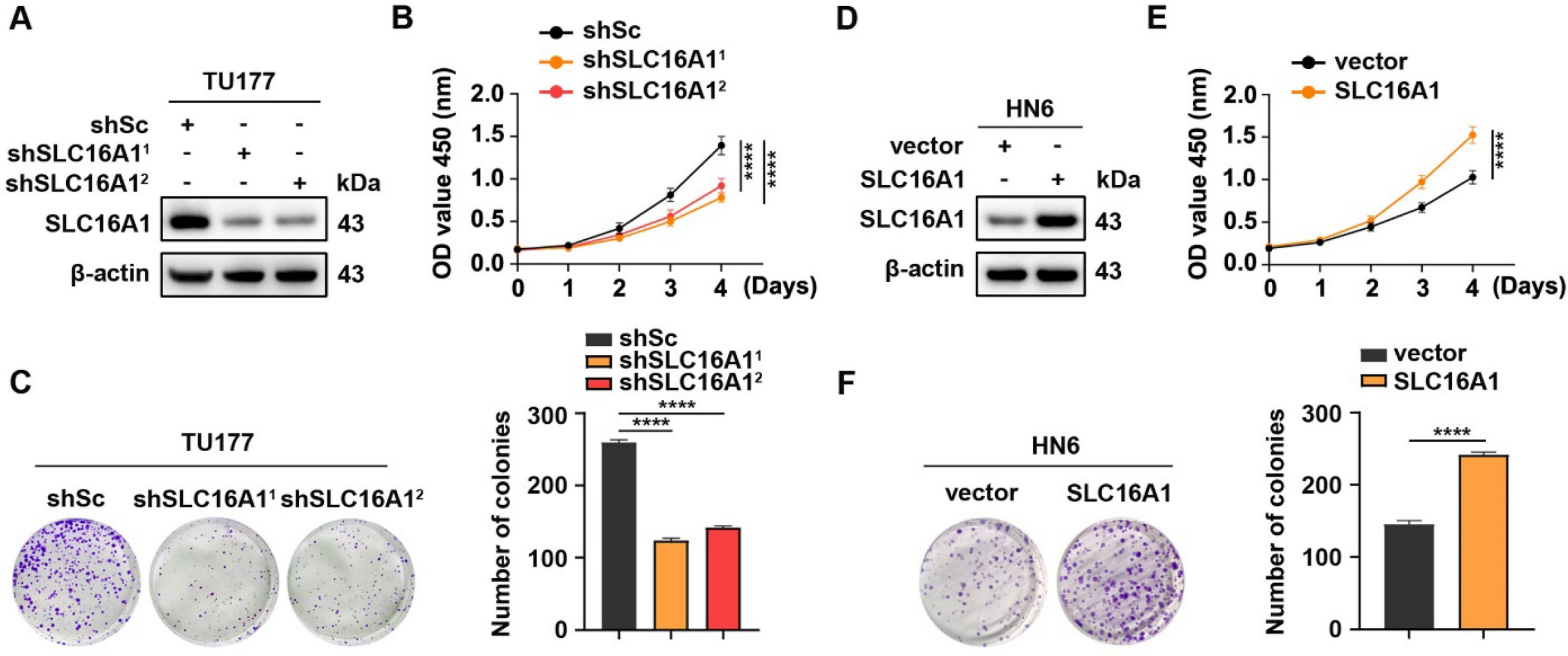
** SLC16A1 promotes HNSCC cell proliferation.** (A-C) TU177 cells were transduced with lentivirus expressing shRNAs against SLC16A1 (shSLC16A1^1^ and shSLC16A1^2^) or a scrambled sequence (shSc). (D-F) HN6 cells were infected with control (vector) lentiviruses or lentiviruses encoding SLC16A1. (A-F) The protein expression level of SLC16A1 was assessed by western blotting (A and D); the cell proliferation was evaluated by CCK-8 (B and E) and colony formation (C and F) assays. Representative images (left panels) and quantifications (right panels). Error bars indicate mean±SD of triplicate samples. ****P<0.0001.

To elucidate the biological function of SLC16A1 in HNSCC, we used two short hairpin RNAs (shRNAs) to knockdown SLC16A1 in TU177 cells, which highly expresses this gene **(Figure 4A)**. CCK-8 assays and colony formation experiments demonstrated that the knockdown of SLC16A1 significantly inhibited the proliferation and colony-forming abilities of TU177 cells **(Figure 4B-C)**. In addition, we performed an exogenous overexpression experiment of SLC16A1 in HN6 cells, which have low endogenous expression of this gene **(Figure 4D)**. By performing CCK-8 assays and colony formation experiments, we observed, in contrast, that overexpression of SLC16A1 significantly enhanced the proliferation and colony-forming abilities of HN6 cells **(Figure 4E-F)**. These results suggest that SLC16A1 plays a critical role in the proliferation of HNSCC cells.

### SLC16A1 promotes HNSCC cell migration and invasion

**Figure 5:**
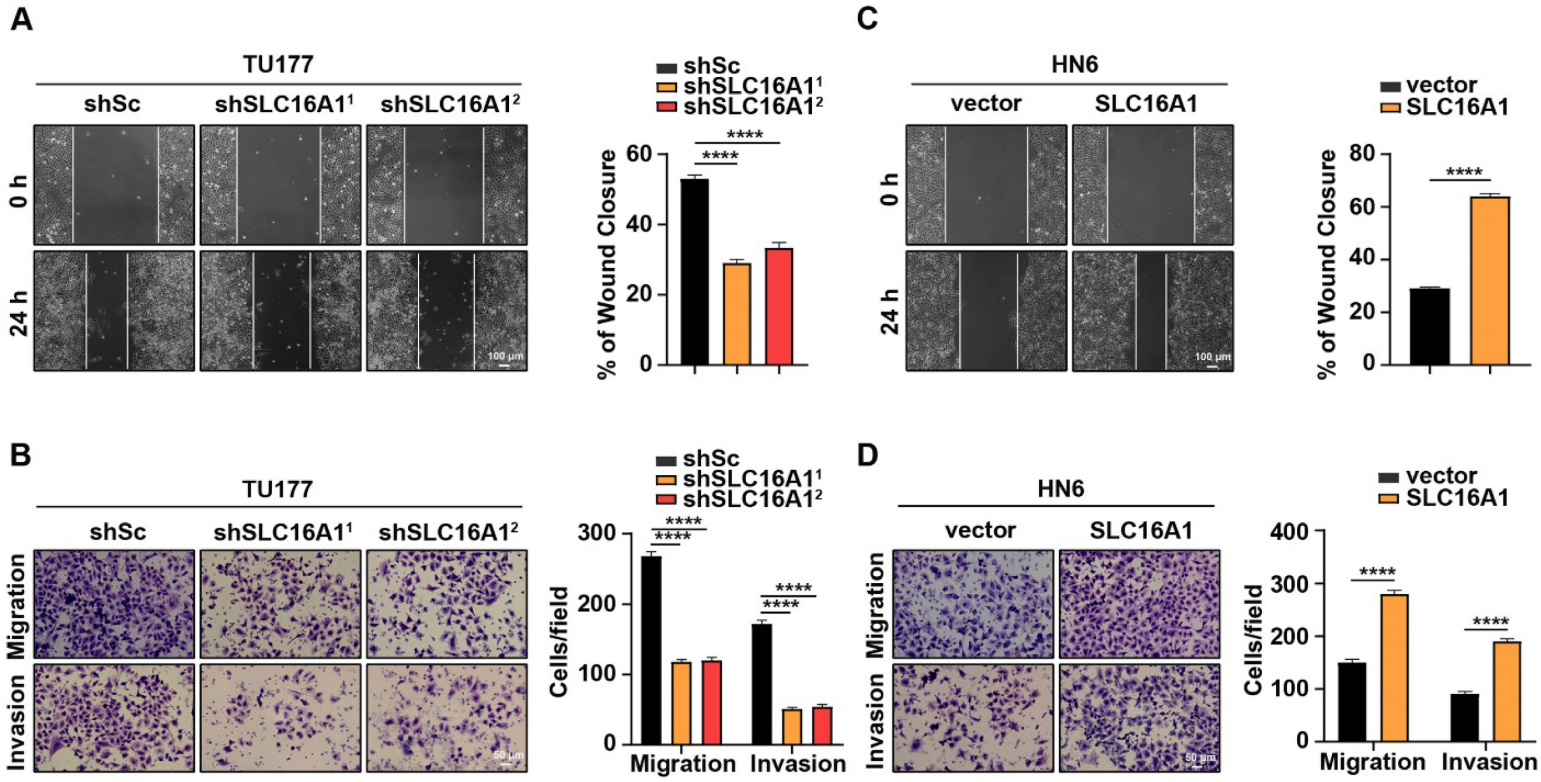
** SLC16A1 promotes HNSCC cell migration and invasion.** (A-D) The migration and invasion rates in SLC16A1-knockdown TU177 cells and SLC16A1-overexpressing HN6 cells were tested using the wound healing assay (A and C), Scale bar, 100 μm, and the transwell assays (B and D), Scale bar, 50 μm. Representative images (left panels) and quantifications (right panels). Error bars indicate mean±SD of triplicate samples. ****P<0.0001.

Given that migration and invasion are pivotal characteristics of tumor development and progression, we conducted *in vitro* migration and invasion assays using HNSCC cell lines with varying levels of SLC16A1 expression. We initially used wound healing and Transwell assays with TU177 cells in which SLC16A1 and their respective controls had been knocked down. Notably, compared with the control cells, the migration and invasion abilities of TU177 cells were significantly reduced following the knockdown of SLC16A1 **(Figure 5A-B)**. Conversely, in HN6 cells where SLC16A1 was overexpressed, the migration and invasion capabilities were notably augmented compared with the control cells **(Figure 5C-D)**.

### SLC16A1 expression level regulates the sensitivity of HNSCC cell to the ferroptosis inducer RSL3

**Figure 6:**
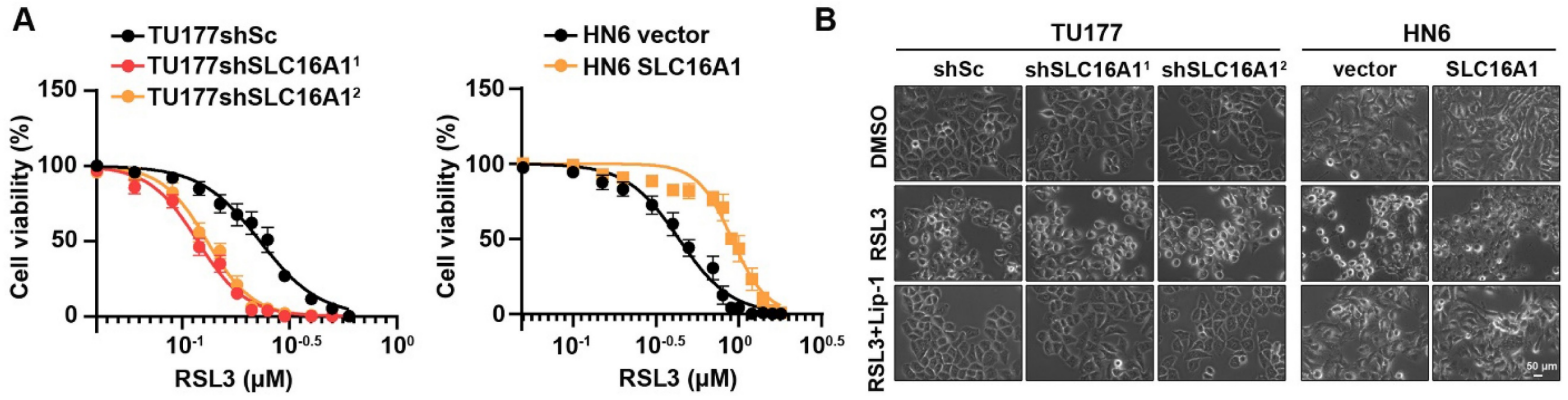
** SLC16A1 expression levels affect sensitivity to the ferroptosis inducer RSL3.** (A) Cell viability of the indicated cells following treatment with RSL3 for 24 h. (B) The indicated cells were treated with RSL3, either in the presence or absence of Lip-1, for 24 h. The corresponding phase contrast images are shown. Scale bar, 50 μm.

Prior study, has reported that SLC16A1 plays a role in resisting ferroptosis 27, but the specific mechanisms by which it does so in HNSCC remain unclear. We hypothesized that SLC16A1 may promote the proliferation of HNSCC cell lines by inhibiting ferroptosis. To test this hypothesis, we conducted drug sensitivity assays using the ferroptosis inducer RSL3 in TU177 cells (with SLC16A1 knocked down) and their control cells, as well as in HN6 cells (with SLC16A1 overexpressed) and their respective control cells. The results revealed that the knockdown of SLC16A1 in TU177 cells significantly increased their sensitivity to the ferroptosis inducer RSL3 compared with control cells. Conversely, the overexpression of SLC16A1 in HN6 cells significantly reduced their sensitivity to the ferroptosis inducer RSL3 compared with control cells** (Figure 6A)**. Furthermore, we observed that the knockdown of SLC16A1 exacerbated RSL3-induced cell death in TU177 cells, whereas the overexpression of SLC16A1 alleviated RSL3-induced cell death in HN6 cells **(Figure 6B)**. Notably, RSL3-induced cell death could be rescued by the ferroptosis inhibitor Lip-1** (Figure 6B)**.

### SLC16A1 facilitates resistance to ferroptosis

**Figure 7:**
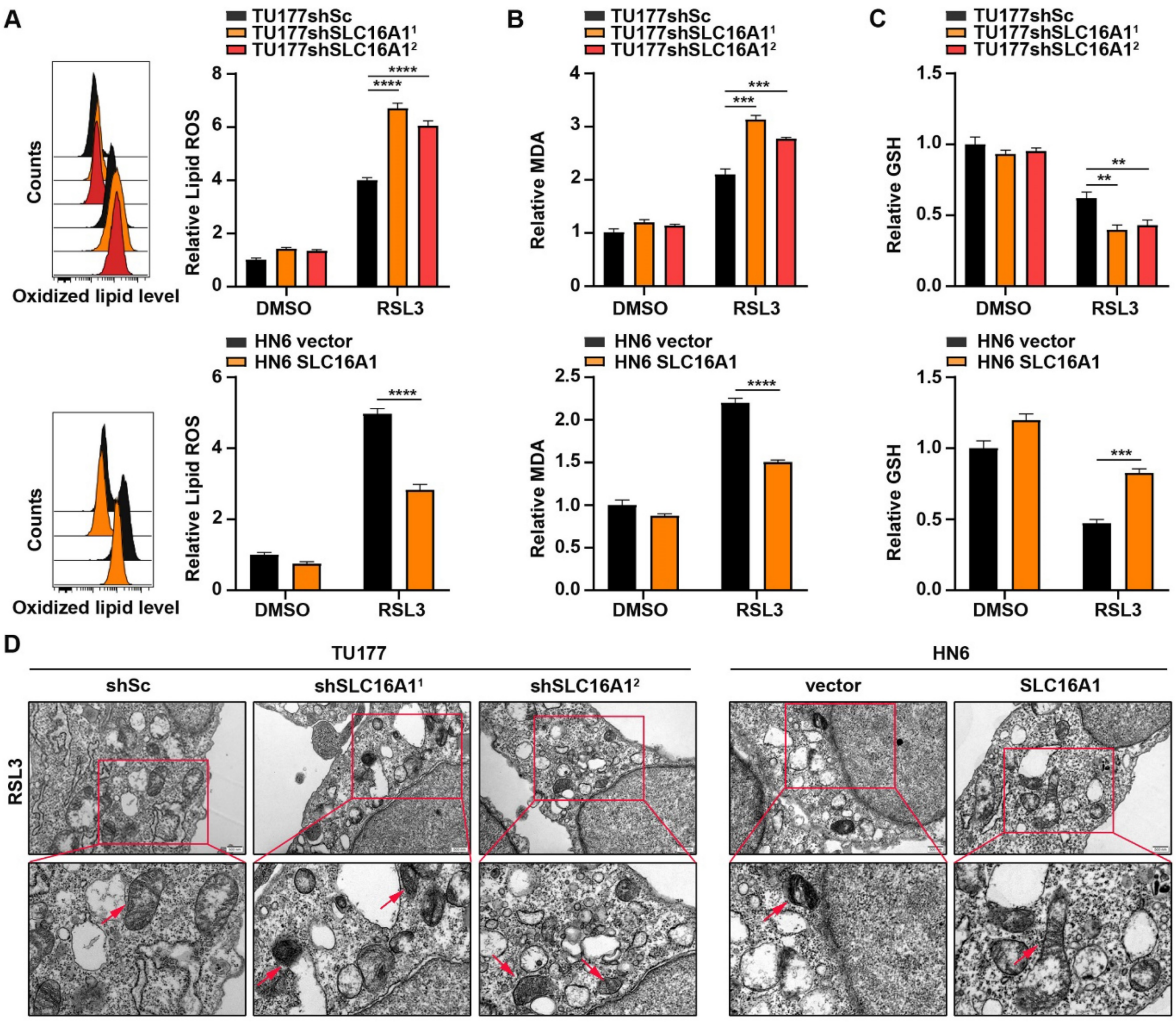
** SLC16A1 facilitates resistance to ferroptosis.** (A-C) The indicated cells were treated with or without RSL3 for 24 h, and then L-ROS (A), MDA (B), and GSH (C) were assayed. (D) Representative TEM images of the mitochondrial morphology in the indicated cells treated with RSL3 for 24 h. Scale bar, 500 nm. Error bars indicate mean±SD of triplicate samples. **P<0.01; ***P<0.001; ****P<0.0001.

To further demonstrate the protective role of SLC16A1 against ferroptosis in HNSCC cell lines, we used BODIPY-C11 to detect lipid peroxidation levels in cells with SLC16A1 knockdown or overexpression. As shown in **Figure 7A**, knockdown of SLC16A1 exacerbated RSL3-induced lipid peroxidation, whereas overexpression of SLC16A1 significantly reduced lipid peroxidation levels. Accordingly, knockdown or overexpression of SLC16A1 also altered the levels of MDA** (Figure 7B)**.

Ferroptosis is a novel form of cell death resulting from iron-dependent lipid peroxidation that is closely related to the levels of GSH within the cell. Therefore, we next explored the relationship between SLC16A1 expression levels and GSH content. As shown in **Figure 7C**, knockdown of SLC16A1 led to a significant decrease in intracellular GSH levels, whereas overexpression of SLC16A1 resulted in a significant increase in GSH levels. In addition, transmission electron microscopy showed that SLC16A1 knockdown cells treated with RSL3 displayed shrunken mitochondria and increased membrane density, whereas SLC16A1 overexpression cells treated with RSL3 showed markedly improved mitochondrial morphology **(Figure 7D)**.

### SLC16A1 promotes HNSCC tumor growth *in vivo*

**Figure 8:**
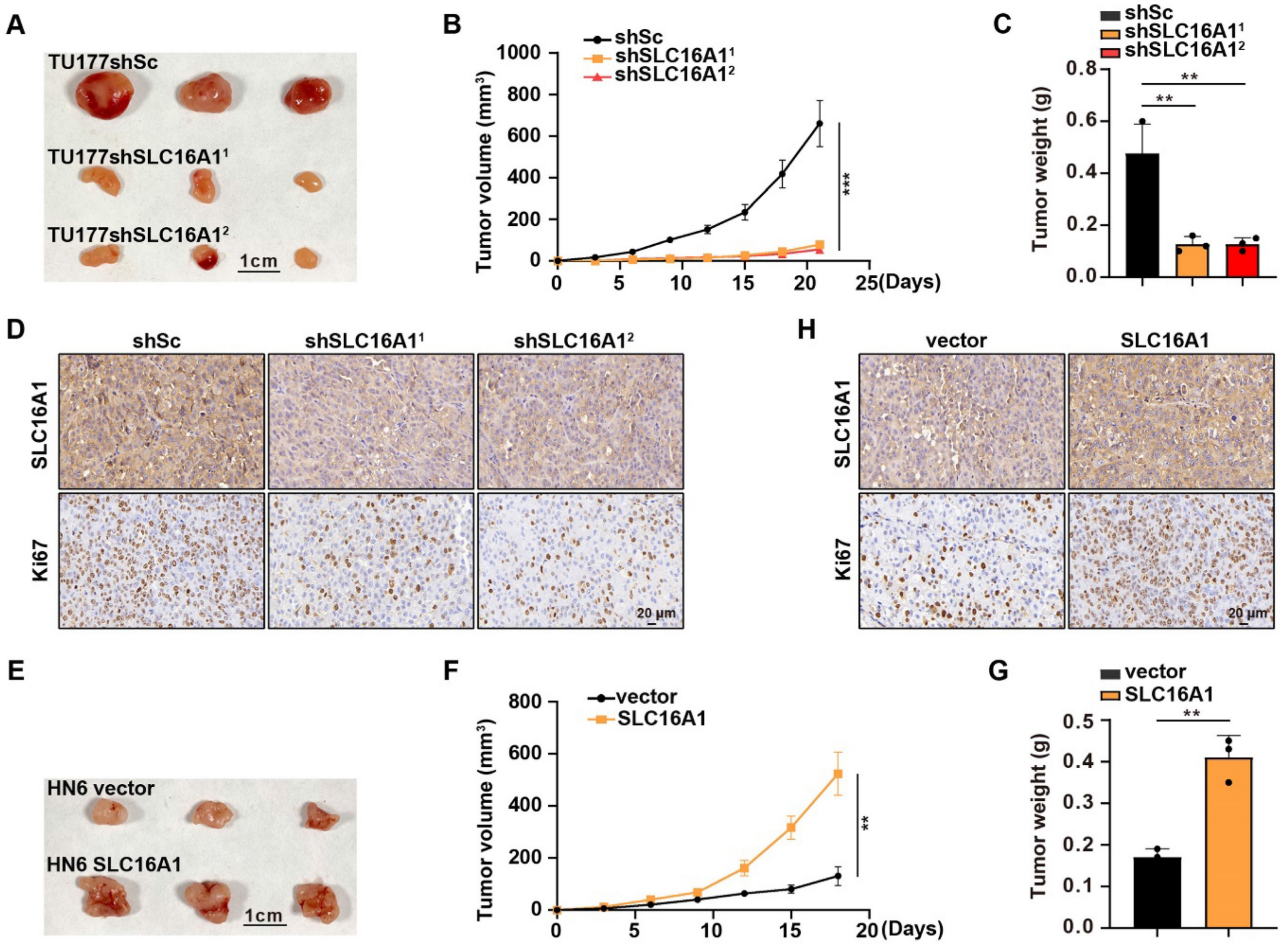
** SLC16A1 promotes HNSCC tumor growth *in vivo*.** (A-H) Tumor growth in mice after the subcutaneous injection of the indicated cells. N=3 for each group. (A) and (E) Tumor pictures, Scale bar, 1 cm. (B) and (F) Tumor growth curves. (C) and (G) Tumor weight. (D) and (H) Representative IHC staining for SLC16A1, Ki-67 of the indicated tumor tissues. Scale bar, 20 μm. Error bars indicate mean±SD of triplicate samples. **P<0.01; ***P<0.001.

Thus far, in this study, we confirmed that SLC16A1 can promote the proliferation of HNSCC cell lines **(Figure 4A-F)**. To further validate the tumor growth-promoting effect of SLC16A1 *in vivo*, we performed a xenograft experiment by subcutaneously injecting SLC16A1-knockdown cells and control cells into the right anterior axillary region of nude mice. The experimental outcomes revealed that mice injected with SLC16A1-knockdown cells exhibited significantly reduced tumor volumes and weights compared with those injected with control cells** (Figure 8A-C)**. Furthermore, IHC analysis of the tumor tissues demonstrated a marked decrease in the expression of the cell proliferation marker Ki-67 in the SLC16A1-knockdown tumors **(Figure 8D)**. Conversely, a xenograft experiment using HN6 cells overexpressing SLC16A1 confirmed that overexpression of SLC16A1 enhanced the proliferation of tumor cells *in vivo*** (Figure 8E-H)**. Thus, our findings indicate that high expression of SLC16A1 positively regulates HNSCC tumor growth.

## Discussion

In recent years, an increasing amount of research evidence has indicated that the SLC16A1 gene exhibits abnormally high levels of expression in various human malignancies and plays a crucial role in promoting the occurrence and development of tumors 21-26. For instance, Huang *et al.* found that downregulating the expression of SLC16A1 through RNA interference technology effectively inhibited the proliferation of cholangiocarcinoma cells and enhanced their sensitivity to the commonly used chemotherapeutic drug 5-fluorouracil 33. Similarly, by constructing a SLC16A1 knockout model, Wang *et al.* observed that this operation significantly suppressed the growth and development of non-small cell lung cancer 34. Despite these findings, the specific mechanisms by which SLC16A1 functions in HNSCC remain underexplored. To address this gap, our study integrated bioinformatics analysis with clinical patient samples to reveal that SLC16A1 is highly expressed in HNSCC and that higher expression levels are strongly correlated with poorer patient outcomes. Furthermore, through a series of carefully designed *in vitro* cellular experiments and *in vivo* animal model experiments, we not only verified that the upregulation of SLC16A1 expression can significantly promote the proliferation, migration, and invasive capabilities of HNSCC cells but also clarified the role of this gene as a key oncogenic factor in HNSCC.

Inducing ferroptosis in tumor cells holds significant promise in cancer therapy; however, the underlying regulatory mechanisms of ferroptosis induction have not yet been fully elucidated. SLC16A1, a protein primarily responsible for the rapid transmembrane transport of monocarboxylates such as pyruvate or lactate, plays a vital role in various cellular physiological processes 19, 20. Prior research by Zhao *et al.* demonstrated that SLC16A1 promotes hepatocellular carcinoma development by inhibiting ferroptosis 27, indicating that SLC16A1 might play a critical role in regulating ferroptosis. In this study, we successfully achieved stable knockdown of SLC16A1 in TU177 cells using lentivirus-mediated technology. The experimental results showed that compared with the control group, the knockdown of SLC16A1 significantly increased the sensitivity of TU177 cells to the ferroptosis inducer RSL3, accelerating both the lipid peroxidation process and ferroptosis triggered by RSL3. Conversely, in HN6 cells overexpressing SLC16A1, the effect of RSL3-induced ferroptosis was markedly reduced. Furthermore, treatment with the ferroptosis inhibitor Lip-1 partially reversed the cell death caused by SLC16A1 knockdown, further confirming the role of SLC16A1 in resisting ferroptosis. Consequently, based on the experimental results, this study not only elucidated the relationship between SLC16A1 and ferroptosis, particularly its mechanism of action in HNSCC but also clarified that SLC16A1 promotes the development of HNSCC by resisting ferroptosis. These findings support prior research into the role of SLC16A1 in tumor biology as well as provide a theoretical basis for developing new therapies targeting SLC16A1.

The tumor microenvironment (TME) plays a crucial role in the initiation and progression of tumors 35-39. Research has shown that SLC16A1 is closely associated with metabolic regulation within the TME, which significantly influences tumor formation and development 40, 41. However, we have not yet delved deeply into this aspect in our research, and future work needs to further explore the interactions between SLC16A1 and the TME and their effects on HNSCC. Nonetheless, our data clearly demonstrate that SLC16A1 is a key oncogene in the occurrence and development of HNSCC. We have not only elucidated the specific mechanisms by which SLC16A1 promotes HNSCC progression through resistance to ferroptosis but also identified a close association between its expression levels and poor prognosis in patients with HNSCC. Despite these findings, our study has certain limitations primarily related to the analysis methods. Specifically, the bioinformatics analysis methods used may not have been sufficiently comprehensive, and the exploration of potential molecular signaling pathways involving SLC16A1 in HNSCC may not have been sufficiently in-depth. Future scientific research should focus on overcoming these limitations to achieve a more comprehensive understanding of the central role of SLC16A1 in the pathogenesis of HNSCC. Overall, the findings of this study provide deep insight into the important role of SLC16A1 in the pathological processes of HNSCC and reveal that the SLC16A1 mediated ferroptosis resistance and tumor growth, and combining SLC16A1 inhibition with ferroptosis-inducers is an effective therapeutic strategy for patients with HNSCC. This discovery has the potential to drive the development of new therapeutic approaches, opening up new avenues for the treatment of patients with HNSCC.

## Supplementary Material

Supplementary table 1.

Supplementary table 2.

## Abbreviations

HNSCC: head and neck squamous cell carcinoma
GSH: glutathione
MDA: malondialdehyde
RSL3: RAS-selective lethal 3
Lip-1: liproxstatin-1
SLC16A1: solute carrier family 16 member 1
DMSO: Dimethyl sulfoxide
TU177: human laryngeal squamous cell carcinoma
NOK: human normal squamous epithelial cells
TU212: human laryngeal cancer cells
FaDu: human pharyngeal squamous cancer cells
HN6: human tongue squamous cell carcinoma
ANM: adjacent normal mucosal
qRT-RCR: quantitative real-time polymerase chain reaction
CCK8: Cell Counting Kit-8
L-ROS: lipid reactive oxygen species
TEM: transmission electron microscopy
IHC: immunohistochemistry
TCGA: The Cancer Genome Atlas
DEGs: differentially expressed genes
shRNA: short hairpin RNA
TME: tumor microenvironment
