# Supplementary material for: SLC16A1 Inhibits Ferroptosis and Promotes the Progression of Head and Neck Squamous Cell Carcinoma
Source: J Cancer. 2025 Mar 29;16(7):2184–96. doi: 10.7150/jca.110217 (PMC12036106; doi:10.7150/jca.110217)
Supplement: Supplementary file 1 — Supplementary table 1. [file jcav16p2184s1.pdf]

Supplementary Table S1. Clinical characteristics of 12 HNSCC patients.

Gender	Age	TNM Stage
Male	73	T1 N0 M0
Male	72	T1 N2 M0
Male	60	T2 N1 M0
Male	59	T1 N1 M0
Male	73	T4 N2 M0
Male	79	T4 N0 M0
Male	72	T4 N1 M0
Male	50	T3 N1 M0
Male	60	T2 N0 M0
Male	61	T1 N0 M0
Male	80	T2 N2 M0
Female	65	T3 N1 M0
